# Replicability of simulation studies for the investigation of statistical methods: the RepliSims project

**DOI:** 10.1098/rsos.231003

**Published:** 2024-01-17

**Authors:** K. Luijken, A. Lohmann, U. Alter, J. Claramunt Gonzalez, F. J. Clouth, J. L. Fossum, L. Hesen, A. H. J. Huizing, J. Ketelaar, A. K. Montoya, L. Nab, R. C. C. Nijman, B. B. L. Penning de Vries, T. D. Tibbe, Y. A. Wang, R. H. H. Groenwold

**Affiliations:** ^1^ Department of Clinical Epidemiology, Leiden University Medical Centre, Leiden, The Netherlands; ^2^ Department of Biomedical Data Sciences, Leiden University Medical Centre, Leiden, The Netherlands; ^3^ Department of Epidemiology, Julius Center for Health Sciences and Primary Care, University Medical Center Utrecht, University Utrecht, Utrecht, The Netherlands; ^4^ Department of Psychology, York University, Toronto, Ontario, Canada; ^5^ Methodology and Statistics Unit, Institute of Psychology, Leiden University, Leiden, The Netherlands; ^6^ Department of Methodology and Statistics, Tilburg University, Tilburg, The Netherlands; ^7^ Netherlands Comprehensive Cancer Organisation (IKNL), Utrecht, The Netherlands; ^8^ Department of Psychology, University of California, Los Angeles, CA, USA; ^9^ Department of Psychology, Seattle Pacific University, Seattle, WA, USA; ^10^ TNO (Netherlands Organization for Applied Scientific Research), Expertise Group Child Health, Leiden, The Netherlands; ^11^ Department of Psychology, University of Toronto, Toronto, Ontario, Canada

**Keywords:** replication, simulation studies, statistical methods, open materials

## Abstract

Results of simulation studies evaluating the performance of statistical methods can have a major impact on the way empirical research is implemented. However, so far there is limited evidence of the replicability of simulation studies. Eight highly cited statistical simulation studies were selected, and their replicability was assessed by teams of replicators with formal training in quantitative methodology. The teams used information in the original publications to write simulation code with the aim of replicating the results. The primary outcome was to determine the feasibility of replicability based on reported information in the original publications and supplementary materials. Replicasility varied greatly: some original studies provided detailed information leading to almost perfect replication of results, whereas other studies did not provide enough information to implement any of the reported simulations. Factors facilitating replication included availability of code, detailed reporting or visualization of data-generating procedures and methods, and replicator expertise. Replicability of statistical simulation studies was mainly impeded by lack of information and sustainability of information sources. We encourage researchers publishing simulation studies to transparently report all relevant implementation details either in the research paper itself or in easily accessible supplementary material and to make their simulation code publicly available using permanent links.

## 1. Background

Many fields of empirical research rely on statistical data analysis. The value of the results of such studies depends on the validity of the statistical methods being used [1]. Under strict assumptions and for relatively simple methods it is possible to mathematically derive how statistical methods will behave when applied to real data, e.g. whether type I error rates are correct or to what extent a method is able to identify an association if it truly exists [2]. However, for more complex research scenarios or more complex methods, the performance of a statistical method is usually assessed by means of statistical simulation studies [3,4]. Simulation studies are computer experiments in which synthetic datasets are generated using computer algorithms [3–5]. A key feature of these experiments is that the mechanism by which the data are generated is known and can, therefore, serve as a benchmark against which methods are compared. In addition, the flexibility of simulation studies in changing the data-generating mechanism means that methods can be tested under various conditions, such as different sample sizes, numbers of variables, and relations between variables.

Results of simulation studies often have a major impact on the way empirical research is conducted and analysed. A striking example is the simulation study performed by Peduzzi *et al*. (cited >7900 times on Google Scholar, April 2023) on the sample size required to fit a logistic regression model, which is one of the most commonly used statistical models in the biomedical sciences [6]. This simulation study has had a major impact and even led to a widely used rule of thumb: the ‘one-in-ten rule’. However, this rule could not be replicated in a replication study by van Smeden *et al*. [7], suggesting that the results of the former study might not be as generalizable as its high citation count might indicate.

Although simulation studies are a powerful tool for methodological research, results from those studies, as the example of the ‘one-in-ten rule’ illustrates, are not definitive. Like empirical results, results from simulation studies need to be reproduced and replicated to verify their veracity [2], and this is increasingly called for [8–10]. So far, there is limited evidence on the reproducibility or replicability of simulation studies. Therefore, we aimed to investigate the extent to which highly cited simulation studies could be replicated. The present study did not seek to improve upon nor criticize the original authors' approaches.

The current study aims at the following:

— discussing the definitions of reproducibility and replicability in the context of simulation studies;
— illustrating that replicability of simulation studies is not a given, using the replication of eight simulation studies as an example;
— describing features that hinder and facilitate replicability of the original studies; and
— providing preliminary recommendations for future simulation studies to facilitate replicability, in addition to available guidance for reporting of simulation studies.

### 1.1. Reproducibility and replicability of simulation studies

There is no broad agreement on what the term ‘replicability’ means in the context of simulation studies [11–14]. For the purposes of this work, we rely on terminology defined in *The Turing Way* [15] and extend it to consider the defining characteristics of reproduction and replication in simulation studies (table 1).

**Table 1.** Implementation of reproduction and replication in empirical studies and simulation studie*s*.

	definition by *The Turing Way*^a^	implementation in empirical study	implementation in simulation study
reproduction	producing the same results using the same data and performing the same analysis	applying original analysis scripts to original data	applying original analysis scripts to original data directly or to data that was newly generated using the original script
replication	producing similar results using different data and performing the same analysis	collecting and analysing new data, following procedures in the original study as closely as possible	writing new code to generate and analyse new data, following procedures in the original study as closely as possible

^a^The Turing Way. 2020 Definitions for Reproducibility. Aspects defined by *The Turing Way* that are omitted from the current table are robustness (same data, different analysis) and generalizability (different data, different analysis). Available from: https://the-turing-way.netlify.app/reproducible-research/overview/overview-definitions.html [Accessed October 2023].

Reproducibility is defined as generating the exact same results using the exact same data and the exact same analysis [15]. Reproducibility in empirical research might look like applying analysis scripts that are available to analyse the original data to evaluate if results are the same as what is presented in the published paper. All research should, as a bare minimum, be reproducible, and failures to reproduce the results of a study suggest there could be an error or some other issue with the study that would reduce its value [16]. However, successful reproduction of a study does not add additional evidential weight [17]. Some have suggested that studies which are not reproducible should not be considered as candidates for replication, because the results of such a replication would be difficult to interpret in the absence of reproducibility [18]. Reproducibility studies of empirical research have varied in their success rate, but none have been completely successful [19–26].

In the context of simulation studies, we believe it is important to extend the definition of reproducibility slightly from considering the exact same data to include the exact same *data-generating process*^1^[27]. For example, if (open) code for a simulation study is available but the original study did not set the random seed as part of the analysis, then the exact same data cannot be recovered, but we believe that such a case falls squarely within the purpose of reproducibility. To consider an equivalent case for empirical research, there are many analysis strategies that rely on random-number generation (e.g. bootstrapping, EM algorithms, multiple imputation), so considering a study with open data and open code but with no seed set for the analysis, the exact same results may not occur because of the randomness in the analysis. Still, though, we believe that this process fits the purpose of reproducibility. Additionally, permitting the use of different seeds allows for potential detection of ‘seed hacking’, i.e. research teams using outlier seeds that result in more extreme results than most other seeds [28]. Reproducibility should be a minimum standard for simulation studies: providing open data and code poses no ethical barriers and thus should be required for all published simulation studies.

Replicability for empirical data is defined as conducting the same analysis with different data collected using methods as similar as possible to the original study and obtaining a similar result [15]. Even in an ideal world, we would not expect all research to replicate, because type I and type II errors are probablistically defined. A failure to replicate the results of a study might call into question the broader theory supported by the findings [16]. Alternatively, successful replication would provide additional evidential weight to the claims from the original study [17].

In the context of simulation studies, replicability involves writing new code to generate and analyse data, following the procedures of the original study as closely as possible. This is in contrast to reproducibility, where the code or data of the original study is used. The ease with which procedures from the original investigation can be followed depends on the granularity of the implementation details available. Apart from a high-level summary in manuscript text, simulation studies can include supplemental material with detailed technical information and even the original code. If new code for a replication study is written by more or less retyping the original code, it is clear that this would hardly add any evidential value and that the replication attempt would be closer to a reproduction. On the other hand, performing replications without using the original code has been advocated because the goal should be that independent and reasonably expert researchers can implement the same procedure^2^ [29,30]. We believe that in a replication the original code can be consulted, but that the replication should in principle be an attempt to write independent code for data generation and analysis. In addition, it is not a given that availability of code guarantees understanding of the original procedures. Code is often not self-explanatory and so integrating it into independently generated new code can be challenging [31].

A similar distinction in approach and goals of reproducibility and replicability is differentiating between ‘computational reproducibility’ and ‘independent *reproducibility*’ [26]. As previously indicated, our work uses terminology defined in *The Turing Way* [15].

## 2. Methods

The focus of our replicability assessment was whether the original description of the simulation was understandable to the replicator(s) and the degree to which they were able to implement it. The primary outcome was to determine feasibility of recreating experimental conditions corresponding to the original studies. Equivalence of results found between the original study and the replication was used as a means to assess replicability, i.e. we assumed that results turn out similar if simulated data, implemented computations, and software functionalities are similar [17,32]. We believe this to be a reasonable assumption for computational research and thus that a focus on feasibility is relevant (assessment of feasibility is explained further below).

### 2.1. Selection of studies

To investigate the replicability of highly cited statistical simulation studies, we identified studies that assessed the performance of statistical methods and are commonly cited within the field of health science or social science. We chose to focus on studies published after 2000 with a high citation count (greater than 1000), because these studies arguably have the largest impact on subsequent empirical studies. We allowed replicators to identify a study of substantive interest to them that met this criterion. An overview of the studies ultimately included can be found in table 2. The number of citations for the included studies ranged from 1650 to 7098 (based on Google Scholar citations retrieved in March 2022). Notably, none of the original simulation studies provided open data or code.

**Table 2.** Statistical simulation studies that were replicated.

authors [reference], *journal*	title	number of citations in Google Scholar (March 2022)	replicators
Austin (2011), *Pharmaceutical Statistics*	Optimal caliper widths for propensity-score matching when estimating differences in means and differences in proportions in observational studies	2265	AL & RHHG
Brookhart *et al*. [34], *American Journal of Epidemiology*	Variable selection for propensity score models	1911	KL & BBLPdV, JCG & FJC
Flora & Curran [35], *Psychological Methods*	An empirical evaluation of alternative methods of estimation for confirmatory factor analysis with ordinal data	2932	YAW & UA
Fritz & MacKinnon [36], *Psychological Science*	Required sample size to detect the mediated effect	3784	JLF & AKM
MacKinnon *et al.* [37], *Multivariate Behavioural Research*	Confidence limits for the indirect effect: Distribution of the product and resampling methods	7098	TDT & AKM
Peters *et al*. [38], *JAMA*	Comparison of two methods to detect publication bias in meta-analysis	1654	AL & RHHG
Rhemtulla *et al.* [39], *Psychological Methods*	When can categorical variables be treated as continuous?	1650	AL & AHJH
Vittinghoff & McCulloch [40], *American Journal of Epidemiology*	Relaxing the rule of ten events per variable in logistic and Cox regression	3031	RCCN, JK & LH

### 2.2. Replication set-up

Teams of replicators retrieved relevant implementation details for the replication from the original publication of their choosing. This information was then used to write simulation code in an open-source programming environment of their choice, with the aim of assessing the feasibility of recreating the experimental conditions that generated the original results. Results of the replication were compared to those reported in the original publication, with the primary outcome of our study being to determine the feasibility of translating the information provided in the original studies into computer code.

#### 2.2.1. Replication teams

Each study was replicated by teams of at least two replicators, consisting of a primary replicator and co-pilot(s). All replicators had formal training in quantitative methodology corresponding to the minimum of an M.Sc. degree in statistics, psychology, or epidemiology. All replicators had prior experience in conducting simulation studies. Replicators extracted information pertaining to the implementation of the simulation studies from the original publication and translated this information into simulation code. The primary replicator coded and ran the replication simulation. The co-pilot(s) studied the simulation code and provided feedback as needed. If feasible, the simulation was run, and results were reported. If not feasible, we report barriers to replication.

#### 2.2.2. Information about the simulation studies

Relevant implementation details for the replications were obtained from the original publications. Information that was explicitly referenced in a publication was also considered. Each team of replicators kept track of information that was ambiguously reported and noted assumptions that they had to make.

#### 2.2.3. Software

The replicators could choose any open-source programming environment for the replication irrespective of the original implementation. All replicators conducted their replications in R statistical software [41]. Details regarding corresponding packages and software versions can be obtained from the individual replication reports (provided in the supplementary materials). For reproducibility of our work, all replication code can be obtained from the project's GitHub organization: https://github.com/replisims/.

### 2.3. Assessment of replicability

Each replicator team aimed to replicate the original simulation study by creating simulation code and performing analyses as identical as possible to the original study, based on the information provided in the manuscript. As the replication of simulation studies is a novel endeavour, there are currently no set criteria to assess the alignment of replicated simulation results with the original results. All factors hindering or facilitating the process were documented by the replicators, because these factors can provide valuable insights for the improvement of future simulation studies. Agreement between results from the replication studies and the original studies was assessed in a qualitative manner and involved evaluating: whether numerical values from the replication studies were comparable to those in the original studies, whether trends in the results were moving in the same direction, and whether the performance rankings of different simulation scenarios matched those in the original studies. Replicators did not check for appropriateness of applied methods or correctness of the original methods.

While we focused on the information provided in the original publication and supplemental materials, the original authors were contacted after the replication attempt was finished as a means to assess the accessibility of possible additional information that could facilitate a replication attempt. The original authors were not contacted earlier to eliminate the possibility of author-provided information influencing the interpretation of the original publication. The authors of each publication were contacted via email with a request for additional information or computer code pertaining to their simulation study. In the case of no response, a single reminder was sent.

## 3. Results

We begin the results section with an overall summary of how feasible the replication of each study was. Given the low number of replicated studies, we deemed a quantification of findings inappropriate. Instead, we identified features hindering or facilitating replicability of simulation studies by providing examples of the replicators' experiences. The discussion of experiences is narrative rather than systematic, meaning that examples listed are illustrative and not comprehensive. An overview of topics of the original studies and key aspects of replicability per study can be found in table 3. In the supplementary materials, we provide an overview of individual study features that hinder or facilitate replication, as well as replicator degrees of freedom, which we define as the flexibility involved in the process of replicating a (simulation) study [46].

**Table 3.** Experienced facilitators and barriers for replication of statistical simulation studies.

author(s) [reference]	brief description of study aim	overall feasibility of replication	replication facilitators	replication barriers
Austin [33]	to assesses the effect of varying caliper width when using propensity-score matching. Simulated data are tabular and resemble clinical observational studies that estimate differences in means and differences in proportions using propensity-score matching. Performance measures are bias, mean squared error, coverage, and type I error rate	partial	•the magnitude of parameters for data generation was explicitly mentioned	•the computational cost of propensity-score matched samples was high
			•provided formulas made it straightforward to compute the intended performance measures	
Brookhart *et al*. [34]	to illustrate variable selection problems in propensity-score modelling. Simulated data are tabular and resemble clinical observational studies that estimate exposure effects, either by entering the propensity scores in the outcome model or using subclassification	almost perfect	•the original article provided clear descriptions of the simulation methods	•some of the results were presented as figures only, which hampered the exact comparison of results
	performance measures are bias and mean squared error		•provided formulas made it straightforward to implement the intended approach	•specific software implementations were not described clearly, such as how splines were fitted. Although the implementation had to be assumed, results were still replicable
			•the data-generating mechanism was depicted in a figure, which was helpful in understanding the simulation set-up	
			•the replicators were familiar with the literature in this topic. This experience could be used to assume the most likely approach for implicit decisions	
Flora & Curran [35]	to compare estimation methods of confirmatory factor analysis models for ordinal variables: weighted least squares estimation versus robust weighted least squares	partial	•the original article provided most of the theoretical information and instruction required to replicate the study	•the exact values (tau) used to transform continuous data into ordinal data were not reported; tau values for five-category ordinal data referenced by the authors produced distributions inconsistent with the original article, but a correction reported in Chalmers and Adkins [42] resolved the inconsistency and was used in subsequent replications
	simulated data are tabular and resemble social science studies with ordinal observed variables from either a normal or non-normal latent process		•a recent article by Chalmers and Adkins [42] provided the code for a partial replication of the same study using the SimDesign package in R statistical software	•the original study used proprietary software EQS and Mplus, whereas the replication used open-source R statistical software
	performance measures are mean relative bias, and mean and variance of test statistics over iterations			•the links to the technical appendix of the original paper, which was described as containing example code for data generation and model estimation, were broken. The replication team was not able to locate the appendix
Fritz & MacKinnon [36]	to compare the sample sizes required to achieve 80% power from six different tests of the mediated effect at various effect-size combinations. Simulated data are tabular and resemble social science studies investigating mediation effects. The performance measure is the required sample size to obtain statistical significance of the indirect effect	almost perfect	•the original article provided a clear and detailed description of the methods that were implemented	•the criteria for the margin of error on power level for bootstrap simulation methods was not explicitly stated in the article. The assumed margin of error had a small influence on the replicability of results
			•the replicators were familiar with the literature in this topic, including work by the authors on this particular study. This experience could be used to assume the most likely approach for implicit decisions	•one of the methods was originally written in the programming language FORTRAN and was unreadable in any open-source programming language. Instead, the RMediate package in R statistical software was used (Tofighi & MacKinnon [43]). Although many functionalities were similar to the original method, some results could not be replicated because the version of the method written for R statistical software sometimes halted execution because of a bug (known to the authors of the method)
MacKinnon *et al.* [37]	to compare methods of estimating confidence limits for indirect effects in mediation analysis. Simulated data are tabular and resemble social science studies investigating mediation effects. Performance measures are accuracy, type I error rate, and power	partial	•the overall structure of the simulation study was easy to glean from the original article, and the simulation conditions were explicitly laid out	•critical values used in one of the inferential methods included in the original simulation study were obtained from the tables in a book [44]. However, no further information was provided on how the values printed in these tables were implemented in the code for the original simulation study. Consequently, a similar (but different) inferential method that was available in an R package created by the original first author had to be used in the replication instead (Tofighi & MacKinnon [43])
			•although some were unclear, instructions were provided for implementing all methods used in the simulation study	•formulas provided in the original publication to calculate certain statistics used in the inferential methods, such as t-statistics and skewness values, were unclear, and so the original formulas had to be retrieved from a book hidden behind a paywall [45]
			•the replicators were familiar with the literature in this topic, including work by the authors on this particular study. This experience could be used to assume the most likely approach for implicit decisions	•one of the methods was originally written in the programming language FORTRAN and was unreadable in any open-source programming language. Instead, the RMediate package in R statistical software was used (Tofighi & MacKinnon [43]). Although many functionalities were similar to the original method, some results could not be replicated because the version of the method written for R statistical software sometimes halted execution because of a bug (known to the authors of the method)
				•links provided in the original publication that contained code / other information necessary for the replication attempt were broken, or they connected to a website that no longer contained the needed information. As a result, one of the inferential methods examined in the original study had to be dropped from the replication simulation
Peters *et al.* [38]	to compare two regression tests for the detection of publication bias in meta-analyses: Egger's regression test versus an alternative, denoted Peter's regression test in this replication. Simulated data resemble meta-analysis input from original clinical studies under publication bias and effect heterogeneity. Performance measures are type I error rate and power	partial	•data-generating mechanism and simulation scenarios were relatively well described in a technical report (which was not accessible in the public domain)	•the description of some simulation experiments was incomplete. For instance, the combination of sample size and expected event fraction could result in datasets without any events occurring, meaning that subsequential analyses could not be performed. Assumptions had to be made on how to replicate the simulation scenario
			•the technical report presented complete results for all investigated simulation scenarios	•results were presented as figures only, which hampered the exact comparison of results
Rhemtulla *et al.* [39]	to compare estimation methods of confirmatory factor analysis models for categorical variables: robust Maximum Likelihood versus categorical least squares. Simulated data are tabular and resemble social science studies with ordinal observed variables from a latent construct. Performance measures are convergence failures, improper solutions, parameter estimates, parameter bias, and coverage	almost perfect	•the original article contained a well-structured methods section that detailed all simulation experiments in separate sections. It was clear from the descriptions how to implement the method	•while error descriptives allowed for comparison of errors occurring across different software implementation, the error handling was not described in sufficient detail to evaluate the effects of the errors on the results
			•descriptives for the generated data allowed for an easy check of the data-generating mechanism	
			•the original manuscript presented descriptive results of errors that had occurred. This enabled us to compare the number and type of errors that occurred in our replication to the original study	
			•results were presented in tables in the supplementary files	
Vittinghoff & McCulloch [40]	to find situations in which a rule of thumb for sample size of clinical prediction modelling of binary and time-to-event outcomes does not hold. The rule states that 10 events are needed per predictor variable. Simulated data are tabular and resemble clinical prediction studies. Performance measures are coverage, type I error, and relative bias	not at all		•simulation parameters were insufficiently described, hampering replication at an early stage
				•data-generating procedures only contained a description of expected results without specifying the procedure to generate it. For instance, the correlation of a binary predictor with continuous predictors was described, but it was not indicated how the binary predictor data was generated and how the correlation with other variables was introduced

### 3.1. Overall feasibility of replicability

In three studies, almost perfect replication of results was achieved [34,36,39]. For one study, not enough information could be obtained to implement any of the reported simulation scenarios [40].

Replication was partially feasible in four studies. In the replication of the study by Austin [33], data-generating parameter values reported in the original study did not align with the description of the properties of the data. Therefore, it was unclear whether the implemented mechanism was in line with the original simulation. The replication of Flora & Curran [35] led to results that were overall consistent with the original simulation results. There were differences between the replication study and the original study in the rates of improper solutions, and the direction and magnitude of relative bias of the factor loadings, possibly due to the use of different software environments. That is, the described implementation of the statistical method in the original publication could not always be replicated exactly. In the replication of the study by MacKinnon *et al*. [37], the overall conclusions of the original article were replicated. The original article compared nine methods of constructing confidence intervals for the indirect effect in mediation analysis in terms of performance measures like power and type I error rates. In the replication study, the relative performance of these methods largely agreed with the original simulations. However, for some methods it was more difficult to gauge the procedure from the original study. One method (the empirical-*M* method) had to be excluded from the simulation altogether because no way forward was found. The replication of Peters *et al*. [38] yielded results that resembled the general pattern and direction of the original results. However, only part of the simulation results was presented in the original study's main text. Matching the replicated results to the displayed results was challenging, particularly because results were presented as figures only.

### 3.2. Replicability-hindering properties

#### 3.2.1. Missing information about implementation of procedures

The study by Vittinghoff & McCulloch [40] was missing the most information about the implementation of procedures, and this hampered its replication at an early stage. The study aimed to find scenarios in which a rule of thumb for sample size of clinical prediction modelling of binary and time-to-event outcomes does not hold. The rule states that 10 events are needed per predictor variable for sufficient performance. Reporting on the investigated scenarios (i.e. constellations of simulation parameters) was incomplete, which made it infeasible to replicate the set of parameters used to generate simulated data. In an attempt to recreate the set-up, the reported information led the replicators to specify 10 176 scenarios in the first simulation experiment, whereas the original study mentioned 9328 scenarios only (see elelctronic supplementary material, file, Vittinghoff and McCulloch replication report, p. 6–7 for ambiguities in the simulation parameters). The ability to verify the agreement of the replicated simulation with the original study was so low that replication was discontinued.

Sometimes information was missing because other documents that were referred to could not be retrieved. For instance, in Flora & Curran [35], links to the technical appendix, and the data-generation and analysis code from the published paper were broken,^3^ resulting in uncertainties about information not explicitly reported in the original paper (e.g. tau values used in data generation; see table 3 for details). Broken links also made it difficult to implement several methods included in MacKinnon *et al*. [37] and Fritz & MacKinnon [36]. Web addresses given in the original publication of MacKinnon *et al*. [37] were supposed to connect to an algorithm and critical values needed to perform two of the methods, but one link no longer worked and the other led to a website that had since been updated and no longer contained the necessary critical values. As a result, the method requiring knowledge of these critical values (the empirical-*M* method) had to be omitted from the replication simulation.

Replication was also impeded when important information was provided for some methods but not others. This was, for example, the case in the replication of Fritz & MacKinnon [36]. The original study compared the sample sizes required to achieve 80% power from six different tests of the mediated effect at various effect-size combinations. A margin of error on power level of 0.1% was provided for methods that were evaluated in 100 000 simulation iterations; however, the margin of error was not explicitly stated for the methods that were evaluated in 1000 simulation iterations. Applying the margin of error on power of 0.1% was infeasible (e.g. a sample size of 34 could have 79.9% power and 35 could have 80.1% power, and neither are within the margin of error). The replicators ultimately chose to increase the margin of error to 0.5%. Notably, the largest numeric deviations between the original and replication studies were for the methods evaluated under the adjusted margin of error on power levels.

#### 3.2.2. Lack of information about error handling

Descriptives of occurred errors in the original study are necessary to compare whether the occurrence of errors was similar in the replication. In replicating MacKinnon *et al*. [37], a function not used in the original simulation was implemented to calculate one of the confidence interval methods [43], because no alternative way of implementing the method could be gleaned from the original paper. This function produced errors under certain conditions, but the original paper did not discuss whether similar cases were encountered in the original simulation or what was done in such cases. Ultimately, the replicator team decided to rerun those cases, which resulted in 13 264 rerun iterations (being 1.6% of the total number of iterations in the simulation experiments).

Lacking information on checking and handling of runs with non-converged or inadmissible solutions (e.g. solutions with negative variance estimates) was another barrier to replication. In the replication of Flora & Curran [35], the rate of non-convergence was higher in some of the conditions than that reported in the original study. This was the case for confirmatory factor analysis models estimated using weighted least squares in settings of small sample sizes, i.e. 100 or 200 observations per simulated data set (see electronic supplementary material, file, Flora and Curran replication report, p. 18 for a full discussion of non-convergence rates). Because fit statistics and parameter estimates could not be obtained from non-converged models, these conditions were excluded from the replication (by omitting the entire condition, not just the iterations that did not converge).

#### 3.2.3. Ambiguous information

Omitting a description of intermediate steps of a procedure sometimes led to uncertainty about how they should be implemented. For example, the study by Vittinghoff & McCulloch [40] contained a description of the correlation of a binary predictor with continuous predictors in the dataset, but it was not indicated how this correlation was introduced.

When studies referred to different sources for information, this information could not always be mapped back to the study. For instance, the study by MacKinnon *et al*. [37] referred to a table in a book for the critical values used in one of the methods they examined in their simulation experiments, yet no further information was provided on how the values in the table were translated for use in the simulation procedure (e.g. how cases that resulted in values not exactly reported in the table were interpolated).

Occasionally, information in different parts of the manuscript contradicted itself. For example, Austin [33] specified each coefficient for the data-generating model. However, implementing the coefficients as specified did not result in the marginal probabilities implied in the original manuscript.

#### 3.2.4. Discrepancies in software

When a study used proprietary software, as was the case for Flora & Curran [35], Fritz & MacKinnon [36], MacKinnon *et al*. [37], and Rhemtulla *et al*. [39], replicability was hindered if this software could not be accessed by replicators. For example, differences between the original Flora & Curran [35] study and its replication attempt in the number of improper solutions at small sample sizes and the directions/magnitudes of some relative bias findings might have been due to the replicators using a different (open-source) software with different default settings or computational strategies than the original.

### 3.3. Replicability-facilitating properties

Although one could simply conceptualize replicability-facilitating factors as abstaining from all the practices we described in the previous section, we would like to highlight specific features that we found made our replication attempts easier and potentially more accurate.

#### 3.3.1. Extensive documentation

Extensive documentation made it easy to understand how the simulation experiment was set up in the original study and thereby facilitated replication. One example of well-structured documentation was provided by Rhemtulla *et al*. [39]. Information about each aspect of the simulation set-up could be easily retrieved from the manuscript. Other examples where the overall structure of the simulation was easy to extract from the original article, and where the simulation conditions were explicitly laid out, were Brookhart *et al*. [34], Fritz & MacKinnon [36], and MacKinnon *et al*. [37].

The study by Brookhart *et al*. [34] provided formulas for the approaches studied as well as a depiction of the data-generating mechanism in a figure. These aspects provided clear guidance for how to set up the simulation experiment and made replication of the study relatively easy.

Journals often allow only limited space for documentation, but a way to share extensive documentation is to present the information elsewhere, as in the technical report that accompanied the study by Peters *et al*. [38].

#### 3.3.2. Availability of software implementation

Availability of (parts of) the simulation code clearly facilitates replication attempts. For example, for the study by Flora & Curran [35], part of the simulation code was available as part of the SimDesign package in R statistical software [42], and this code was generalized for the replication. The methods investigated by Rhemtulla *et al*. [39] were conducted using proprietary software in the original study; however, in the replication, it was possible to use the lavaan package [47], which is complemented by an entire structural equation modeling infrastructure for simulation studies (e.g. simsem [48]). While this package did not provide any of the code used in the original simulation, the infrastructure facilitated the implementation of the methods.

#### 3.3.3. Clear presentation of findings

Presentation of simulation results in tables rather than figures also facilitated the assessment of how well a simulation was replicated, and this was done by both MacKinnon *et al*. [37] and Brookhart *et al*. [34]. We do not wish to suggest that for replicability purposes all figures in simulation studies should be replaced by tables. Rather, the approach by Rhemtulla *et al*. [39] could be taken, where complete results were presented in table form in the supplemental materials.

## 4. Discussion

The present study attempted the replication of eight highly cited simulation studies investigating the performance of data analytical methods. In three studies, almost perfect replication of results was achieved [34,36,39]. Replication was partially feasible in four studies [33,35,37,38]. In one study, replication was hampered early on because not enough information could be obtained to replicate the combination of parameters used to generate simulated data [40]. To the best of our knowledge, this is the first attempt to replicate a set of simulation studies and to provide a formal assessment of factors hindering and facilitating replicability. An overview of the lessons we learned that could help make future simulation studies easier to replicate is given in table 4.

**Table 4.** Lessons learned to make future simulation studies more easily replicable.

simulation aspect	recommendations for future simulation studies	good examples from the current replication study that can help with implementing the recommendation
description of procedures in the manuscript (data generation, analysis, and results aggregation/presentation)^a^	•read through the draft of the manuscript from the perspective of whether the procedure is replicable	•the formulas and a figure of the data-generating mechanism in Brookhart *et al*. [34] provided clarity about the set-up of the simulation experiment
	•if possible, seek feedback from another (independent) researcher on whether the procedure is replicable based on the manuscript text	•the structure of manuscript text in Brookhart *et al*. [34], Fritz and MacKinnon [36], MacKinnon *et al*. [37], and Rhemtulla *et al*. [39] provided clarity about the set-up of the simulation experiment
detailed description of procedures in supplemental material (data generation, analysis, and results aggregation/presentation)	•publish additional material together with the paper at the journal platform	•the supplemental materials of Peters *et al*. [38] and Rhemtulla *et al*. [39] contained complete results in table form
	•use permanent links (DOIs) for separately published documents	
description of error handling	•report how errors and missing values due to non-convergence were handled; these strategies are ideally pre-specified in the protocol of the simulation study	•the technical report in Rhemtulla *et al*. [39] contained descriptives of occurred errors
	•provide descriptives on occurred errors and non-convergence	
availability of software implementation	•make code available using a permanent link (DOI)	•part of the simulation code of Flora & Curran [35] was available in the SimDesign package in R statistical software [42]
	•preferably avoid use of proprietary software	

^a^For recommendations on reporting of simulation studies, we refer to [4,49,50].

Information provided in the original publication (plus accompanying documents) was not always sufficient for replication. Information sources were not always sustainable, which previously proved to be a problem in the field of biostatistics [51]. Reported information being insufficient for replication is in line with the results of a review of reporting practices of simulation studies [4]. This observation is not unique to simulation studies and has also been found in empirical research from the medical and social sciences [52]. We speculate that incomplete reporting is partly due to certain details being considered trivial information by the original researchers (and reviewers). In the case of space restrictions imposed at many journals, what is considered trivial or obvious may not be reported in detail. However, for successful replication by researchers not involved in the original research, a detailed description of the simulation procedure is essential, otherwise the replicator has to make (arbitrary) decisions, which may be a source of discrepancy between results of the original simulation study and its replication. Those arbitrary decisions are part of the ‘replicator degrees of freedom’. What is more, some of the replication studies were performed in a different programming language, where functions might have different default settings. This illustrates that decisions are sometimes made implicitly but might deserve explicit reporting.

It is now increasingly common to accompany a simulation study manuscript with technical details in a supplementary file, or to share the full code for the simulation at the journal or at an alternative openly accessible platform. The selected papers were published between 2004 and 2012 and several did not have extensive additional materials. It is to be expected that technical details and ‘detouring implementations' of a simulation study are occasionally omitted from manuscripts to better convey the main message of the study. As availability of detailed information was a facilitating factor in the replications of the current study, we encourage the practice of sharing additional technical documentation and code. Given the many broken links we encountered, we emphasize that additional materials such as supplements or code are ideally posted in a persistent location with a DOI to ensure long-term availability.

The current work focused on replication of the simulation studies, meaning that we focused on whether similar results could be obtained if data generation and analysis were performed as similar as possible to the original study. For simulation studies, it would also be particularly relevant to assess the generalizability of findings about a method by exploring alternative approaches to testing the same question or evaluating novel conditions. Evaluation of when and how a method is ideally implemented requires a different type of methodological research than developing a new method [53].

Several potential limitations of this study need to be addressed. The original simulation studies chosen for replication in the current study were selected based on topic, number of citations, and expertise of the replicators, and are not likely to be representative of simulation studies in general. Each replication team selected their own simulation studies to replicate as well, which could have led to their particular skill sets and interests influencing the studies they chose. With merely eight simulation studies being replicated, our sample was relatively small. Nevertheless, it provided valuable insights into factors that facilitate or hinder replicability of simulation studies. Also, although the replicators were formally trained in quantitative methodology and experienced in conducting simulation studies, they were not necessarily experts on the exact topics that were investigated in the original simulation studies. Possibly, tacit knowledge about a particular field or method could have enhanced replicability. For instance, the simulations by Fritz & MacKinnon [36] and MacKinnon *et al*. [37] were replicated by researchers who specialize in mediation analysis, and these were two of the more replicable studies. Previous replication initiatives found that replicator expertise was indeed related to the reliability of a replication [54,55].

Similarity of results was used to assess replicability. However, when a replication attempt yielded results similar to the original study, few or no further checks were conducted to see whether implementation was actually similar, whether results were obtained due to coincidence, or whether errors were made but cancelled each other out. In contrast, when results differed from those reported in the original study, the code was scrutinized, and some replication teams programmed several implementations to obtain the original result. In the case of insufficient information being available, replicators had to make informed guesses about, for example, possible values of simulation parameters, since computationally intensive procedures prevent a trial-and-error approach to replication. Finally, it is worth noting that the current replication did not address the design of the simulation study itself; that is, how the original authors operationalized the research question.

The teams sought to replicate, rather than reproduce, results of the original study. It may be worth considering whether reproduction should be a prerequisite for replication of simulation studies; however, none of the eight identified studies had openly available data or code (except Flora & Curran [35], which had broken links), and so evaluation of reproducibility of these studies may not be possible. To illustrate alternative routes to original code, we contacted the corresponding authors of the original studies by email after completion of the attempts to replicate the simulation studies based on the information provided in the original publication. All corresponding authors responded to our emails. In some situations, this led to additional information, including (partial) code used for four of the original simulations. At this point, reproducibility could have been evaluated for those studies, but because the focus of this study was on replicability, this information was not used to alter the replications and hence contacting the original authors did not have consequences for the replication attempts. Clearly, complementing the publication of a simulation study with (publicly) available simulation code would greatly enhance reproducibility and replicability. Journals that publish simulation studies should require code and data to be publicly available, similar to recent pushes in empirical research [56,57]. Future research could identify simulation studies with open code to evaluate reproducibility separate from replicability, as these two characteristics can speak to different properties of the original studies. An example of a reproducibility research initiative is the Ten-Years Reproducibility Challenge hosted by the ReScience C project journal [13,58].

### 4.1. Reproducibility and replicability of simulation studies

Reproducibility and replicability of simulations studies are related but distinct goals, just as they are in empirical research. Reproducibility is closely connected to the use of the original materials (code and/or data). Failure of reproduction, especially if attempts at reproduction result in differing conclusions, means that even given the original ingredients and recipe, identical results cannot be reproduced. Previous researchers have differentiated between process and outcome reproducibility [16]. A failure of process reproducibility refers to a lack of information available to generate the same results, whereas a failure of outcome reproducibility means that all the materials are available but still the same results do not occur. Both types of reproducibility could occur for simulation studies, and we believe that reproducibility should be a minimal standard for simulation.

A replicable study is one where an independent research team could collect or generate a new sample from a similar population or data-generating mechanism using methods as close as possible to the original study, conduct analyses as similar as possible to the original study, and find a similar result. A failure to replicate could occur for many reasons, but a common concern is that there may be specific details of the original study that are not reported in the original manuscript or supplemental materials, yet are key to producing the same results. While we perceive reproducibility to be a minimal standard, we believe that replicability should still be sought after because another reasonably expert research should in principle be able to generate similar results based on information reported in the paper and supplemental materials. A failure of replicability could have implications for whether the scientific community deems the research to be true and accurate.

To facilitate both reproducibility and replicability, transparent and clear information on how the study was conducted should be provided and may be expressed in either code or manuscript text. Existing guidance by Morris *et al*. [4] outlines how to report on main aspects, such as the aim of the simulation study, data-generating mechanism, estimand, methods, and performance measures. Subject-specific guidance on reporting is available as well, for discrete-event simulations in operational research [49] and calibration methods to find parameters for cancer simulation models [50]. Additionally, reporting software-specific features such as definitions of improper solutions and version numbers facilitates assessment of reproduction. As indicated above, what is critical information and what is implicitly considered background knowledge may be hard to assess for researchers themselves. Another challenge is that not every detail of a simulation study can be reported in a manuscript while maintaining readability and given length limitations; however, a standard to strive for is reporting any specific decisions that the researcher believes are tantamount to replicability. This type of description may be aided by including ‘Constraints on Generality’ statements [59] in simulation studies.

Making simulation code publicly available, e.g. in a repository or as an online supplement on a journal's website, is needed to improve replicability and reproducibility of simulation studies. Sharing of simulation code ideally gives readers access to details regarding simulation parameter settings, coding environments and dependencies (including their versions), random-number generator seeds, and implementation of algorithms for data generation, as well as data analysis and presentation of results. While data and code sharing for simulations may seem obvious, there may be case-specific limitations that need to be considered (e.g. open code for complex simulations on supercomputers; what level of detail should be included in open data; complications around using alternative operating systems). An example of preparing code for peer review is the ‘Checklist for Code and Data Supplements’ from the *Biometrical Journal* [60,61].

Future studies on reproducibility and replicability of simulation studies are encouraged. The time needed to complete a replication differed per study and was not recorded but was estimated to be at least 40 hours per replication. Similar to Nuijten *et al*. [18], we believe that evaluation of reproducibility of simulation studies may be a useful first step prior to conducting a time- and resource-intensive replication. For future replicators, we recommend replicating a simulation study that is closely related to a planned research project, as this undertaking could serve as a foundation for the planned study. This effort could be extended by investigating the robustness of findings under different data-generating mechanisms or implementation of approaches, i.e. to evaluate generalizability of findings. Replication of simulation studies could be an educational project for trainees [62]. Large-scale replication projects would provide insight into the replicability rate of simulation studies. Such initiatives would ideally include more recent simulation studies as well, to describe features hindering or facilitating replicability of simulations for which code of the original simulation is openly available. Finally, as mentioned before, it would be particularly relevant for simulation studies to assess the generalizability of findings about a method by investigating alternative approaches to testing the same question or evaluating novel conditions.

## 5. Conclusion

Discussions about replicability of research in the fields of biomedical and social sciences have focused on studies with human participants, where replicability may be impaired by heterogeneity of participants across studies. Such heterogeneity should not affect simulation studies investigating statistical methods, which therefore should be perfectly or near-perfectly replicable. This pioneering study showed, however, that replicability of simulation studies is not a given, and the information provided in the original publication of highly cited and influential simulation studies was often insufficient for complete replication. We encourage researchers who publish simulation studies to transparently report all relevant information and preferably make their simulation code and data publicly available to facilitate future research, including reproduction and replication of their simulation study.

## Data Availability

All analysis code is available from the GitHub Organization: https://github.com/replisims. All replication reports in the electronic supplementary material are also available at the Zenodo Community: https://zenodo.org/communities/replisims/. The data are provided in electronic supplementary material [63].

## Footnotes

1 This definition is in line with Bollen *et al.* [27], which focuses on the same ‘materials’ rather than the same data being used for reproduction.

2 Where we remark that the meanings of the terms ‘reproducibility’ and ‘replicability’ in the field of machine learning are exactly the opposite of the definitions of *The Turing Way* that we use.

3 After completion of the replication, it was discovered that the link from the publisher's website (but not in the published paper) was functional and the partial code could be recovered, but this code was not used in the replication attempt.
